# Spatio-temporal photolysis rate profiles of UV_254_ irradiated toluene

**DOI:** 10.1038/s41598-022-16941-6

**Published:** 2022-07-26

**Authors:** Ahmed S. El-Tawargy

**Affiliations:** Optics Research Laboratory, Physics Department, Faculty of Science, Damietta University, New Damietta City, 34517 Egypt

**Keywords:** Physics, Optics and photonics, Nonlinear optics, Materials for optics

## Abstract

The volatile organic compound (VOC) toluene is irradiated with a 254 nm UV source. The studied sample (1 mL) of toluene is equipped in a sealed quartz cuvette and inserted in one of the Michelson interferometer’s arms. During a UV_254_ irradiation of 1 h, the variation in the toluene’s refractive index profiles are monitored as a movement of Michelson interference fringes. These interferograms are recorded and digitally analyzed to produce their phase map distributions and, hence, reconstructing the refractive index profiles which are expressing the toluene’s photolysis behavior. With increasing the UV_254_ irradiation time, the toluene’s refractive index profiles exhibit both temporal and spatial decrease due to the production of benzyl radicals and the consequent oxidation of these radicals. The spatio-temporal refractive index and photolysis rate profiles of toluene are reconstructed and discussed.

## Introduction

Volatile organic compounds (VOCs) can be found in many places such as the industrial processes of coatings and printings as well as being, cautiously, used in cosmetics and fragrances^1–7^. At room temperature, VOCs have high values of vapor pressure which make them spread easily in the surrounding environment^1,2,8,9^. Most VOCs are hazardous and harmful to the human’s health and are considered environmental pollutants as they cause some environmental problems; e.g. a photo-chemical smog^1,9,10^. Different treatments are followed in order to minimize the emissions of VOCs into the ambient air. Among these methods; thermal-oxidation, bio-filtration and photo-degradation^1,11–13^. Photo-degradation is based on the irradiation of the substance by the UV sources^1,14–19^. The efficiency of the removal rate of pollutants by the UV irradiation, sensitively, depends on the UV energy and dosage, method of irradiation, the ambient temperature, relative humidity, air flow, the mixing of air in the environment and, unquestionably, the VOC’s functional groups^20–27^.

The purely abiotic photolysis of VOCs is a type of photo-degradation takes place due to the cleavage of, at least, one type of the covalent bonds of the molecule exposed to the UV energy^1,28–32^. The VOCs’ molecules absorb the incident energetic UV photons which cause the excitation of the electrons of these molecules. However, if the absorbed energy is sufficiently high (i.e., low UV wavelengths), certain chemical bonds can be broken and the VOC is, then, photo-degraded. For instance, the UV-C (i.e., 100–290 nm with equivalent energies 12.4–4.3 eV) are able to dissociate formaldehyde and toluene molecules as the required energy, to break down the C–C and C–H bonds, lies in this band^11,45,46^. Definitely, this causes an alteration in the physical and the chemical properties of these UV irradiated molecules^20^.

Photochemistry at interfaces (e.g., air/VOC inteface) is an important field of study where photolysis, for instance, is more efficient at the interface compared with the bulk substance^33^. In case of a dissolved material in a VOC, the decrease of the solvent cage’s effect plays an important role in the photolysis effect at the interface. Decreasing the effect of the solvent cage is capable of reducing the recombination rate of the photo-fragments and the photolysis’s quantum yield at the interface is, accordingly, increased^33,34^. Nissenson et al. reported on a high difference (*ca.* 3 orders of magnitude) in the photolysis rate in case of small particles of substances compared to their bulks due to the higher intensity of irradaition at the air/VOC interface^33^. Therefore, the photolysis of pure VOCs at their interfaces is an interesting topic to be studied^29,35,36^. The photolysis rate at a substance’s surface can be utilized to estimate the average photolysis rate along an entire column of this substance^32^. Actually, monitoring of a VOC’s rate of photolysis can be implemented using an interferometric technique for sensing the refractive index variations of a substance as a function of both space and time^37–40^. Interferometric techniques provide the ability to the non-contact, non-destructive and in-situ measurement of the refractive index distributions of nonhomogeneous samples such as a UV photo-degraded VOC suffering induced refractive index variations^41^. This gives much insights about the in-situ spatial and temporal changes in the substance’s chemical and, accordingly, physical properties^16,37–40,42^. Despite being a good indicator on the changes of physical properties, monitoring the refractive index variation can’t identify the chemical variations of the studied material and this is considered as a limitation of the proposed method.

In the presented study, the variations of refractive index profiles of UV_254_ (a UV source of wavelength 254 nm) irradiated toluene are monitored using a Michelson interferometer. Then, the toluene’s photolysis rate profiles are calculated. Michelson interferometer permits a double passing of the optical beam through the sample under study^43^. Therefore, it provides a higher sensitivity of measurements compared with the single pass interferometers, particularly when the automated digital image processing is applied on the interference patterns^5,44^.

## Optical system and theoretical considerations

### Optical system

**Figure 1:**
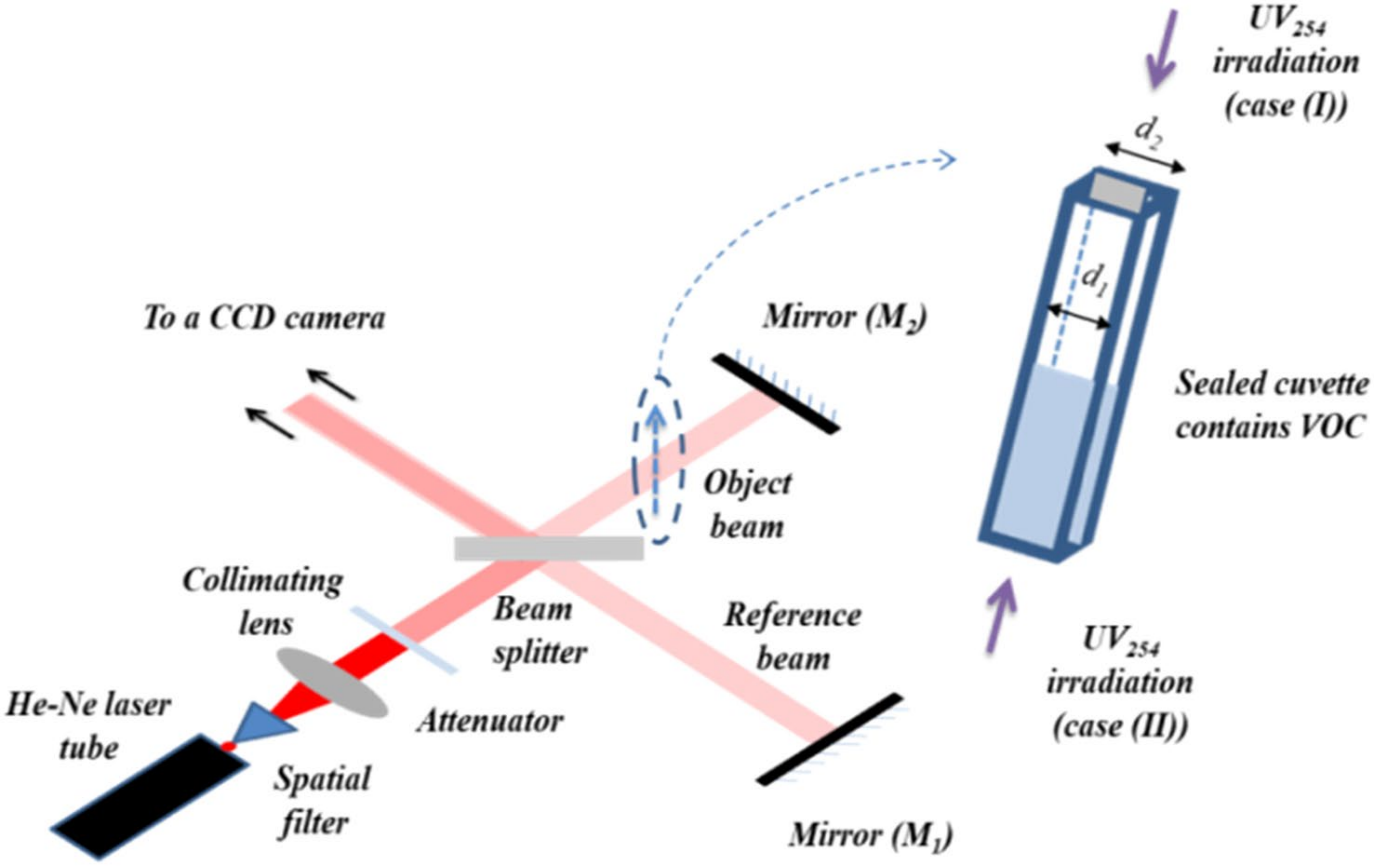
A schematic diagram shows the setup of Michelson interferometer used to study the variations of refractive index profiles of the UV_254_ irradiated toluene.

Figure 1 shows a schematic diagram of the setup of Michelson interferometer used in the presented study. A 10 mW He–Ne laser beam with a wavelength (λ = 632.8 nm) is spatially filtered. Then, a parallel beam is obtained by the aid of a collimating lens. An attenuator is used to minimize the He–Ne laser intensity in order to not optically saturate the imaging camera. The collimated beam’s amplitude is divided into two orthogonal beams (later called, object beam and reference beam) using a beam splitter. These two beams are back reflected using the two mirrors *M*_1_ and *M*_2_ to be recombined by the same beam splitter and producing a two-beam interference pattern. This interference pattern has an intensity distribution (*I*) described by Eq. (1) ^2,43,45^.

1 $$I=4{A}^{2}{cos}^{2}\left(\frac{\delta }{2}\right),$$

where, *A* is the amplitude of each interfered beam, assuming that they are equal, and *δ* is the phase difference between these two interfered beams.

### Theoretical considerations

Suppose a cuvette, contains a transparent sample, is inserted in an arm of a Michelson interferometer (i.e., the object beam) as shown in Fig. 1. The refractive index of the cuvette’s material is (*µ*) and its inner and outer thicknesses are (*d*_1_) and (*d*_2_), respectively, while the sample’s refractive index is (*n*). The optical pathlength of the beam constituting this arm will be increased by a value equals the double of the optical pathlength difference (*OPLD*) where the optical beam passes two times through the sample in case of Michelson interferometer^5,44^. Therefore, a phase change takes place in the interference pattern which is represented as a shifting of a number of fringes (*m*) given by Eq. (2) ^43^. However, the whole interference pattern still has the same intensity distribution of the two-beam interference, described by Eq. (1). Any further stimulated disturbance in the sample’s refractive index can be monitored as a movement of the interference fringes in the field of view.

2 $$m=\frac{2 \times OPLD}{\lambda }=\frac{2((\mu -1)({d}_{2}-{d}_{1})+(n-1){d}_{1})}{\lambda },$$

where, *λ* is the used light’s wavelength (= 632.8 nm, in the presented study).

Under the effect of an external stimulator, the sample’s refractive index will be disturbed (either increased or decreased) by a value (*Δn*). The disturbance causes a change in the optical pathlength and consequently a phase shift which can be observed as a number of fringes (*Δm*) crossing the field of view. *Δm* as a function of *Δn* can be expressed as:

3 $$\Delta m=\frac{2{d}_{1}}{\lambda }\Delta n.$$

Experimentally, *Δm* can be determined by recording two successive Michelson interferograms and reconstructing their phase distributions using a phase reconstruction method; e.g. by employing fast Fourier transformation (FFT). Then, *Δn* can be obtained according to Eq. (3). Obviously, this can be achieved if the rate of refractive index variation is detectable, by the imaging system, as will be explained at the end of “Results and discussion”.

## Methods

### Experimental work

1 mL of toluene (*M*_*m*_: 92.14 g/mol, Purity: 99.9%, purchased from Panreac Química SLU) is dropped in a quartz cuvette which is sealed with a transparent cap in order to keep the studied system closed^8,29^. The quartz is transparent for both visible and UV electromagnetic waves. Therefore, there will be no change in *µ* due to the UV irradiation. The cuvette’s inner dimensions are 10 mm × 10 mm and its outer dimensions are 12.5 mm × 12.5 mm while its height is 40 mm. It is inserted in the shorter arm of the Michelson interferometer, as shown in Fig. 1. After adjusting Michelson interferometer, the mirror *M*_*1*_ is slightly tilted in order to make a small wedge with respect to the other mirror *M*_*2*_ and obtaining the two-beam equidistant straight parallel fringes.

The UV_254_ (4 Watt and a wavelength 254 nm UV lamp supplied by Heraeus Holding, Germany) irradiating source is used to continuously irradiate the sample’s surface for 60 min. Two experimental procedures are performed; when the VOC’s surface is exposed to a stagnant amount of air and when the VOC’s surface is not directly exposed to air as sketched in Fig. 1 (cases *I* and *II*, respectively). In case (*I*), the UV_254_ source is positioned at a distance (*L* = 3 cm) from the studied VOC’s upper surface which is exposed to an abundant amount of oxygen of the stagnant amount (3 cm^3^) of air. In case (*II*), the UV_254_ source is situated at a similar distance below the lower VOC’s surface. In this case, the toluene’s lower surface is not exposed to air. For each experiment, a fresh VOC sample is equipped in the cuvette which is well washed and dried prior to every experiment. Each experimental procedure is repeated two times for the reproducibility purposes. All experiments are performed in a dark ambient of a temperature (27 ± 1) °C and a relative humidity (50 ± 2)%. At these conditions, the toluene’s refractive index is 1.48785 ± (1 × 10^–5^) with the He–Ne laser’s wavelength 632.8 nm.

It is worth emphasizing that the He–Ne laser is used as the probe beam to measure the VOC’s refractive index variations of toluene which is irradiated with the UV_254_ irradiating source. As a controlling experiment, prior to the UV_254_ irradiation, the studied VOC is checked when there is no UV_254_ irradiation and recording that there is no change in the refractive index due to the He–Ne laser over 60 min. In other words, the toluene’s refractive index variations, in the presented study, are exclusively due to the UV_254_ irradiation. See Appendix 1 for more details.

By operating the UV_254_ source for each experiment, the dynamic interference patterns are captured using a CCD camera (ZEISS Axiocam 105, 5 megapixel CMOS sensor) with a frame rate 5 frames/s. This capturing rate is sufficient in this study and enables monitoring the refractive index variations effectively. However, this capturing rate can be increased in case of more rapid variations, i.e., it depends on the rate of refractive index variation. The imaging system is adjusted to image a 5 mm distance of the sample starting from its surface (either upper or lower) towards its interior. The recorded interferograms are analyzed in order to extract their phase distribution maps and then reconstructing the refractive index profiles varying with increasing the time (*t*) of the UV_254_ irradiation. These refractive index profiles are utilized to estimate the rate of photolysis of the studied VOC. In this way and unlike to previous attempts^46^, one doesn’t have to compare the fringes crossing the sample with the fringes crossing the sample-free region. Furthermore, there is no need for the cuvette rotation or any additional mechanical process to optimize certain incidence angles or even to count the moving fringes by the aid of an external fringe counter. The used software of analysis, in the presented study, is prepared to automatically and instantaneously detect and count the fringes’ movement.

### Interferograms’ processing

An interferogram is considered as a 2D matrix of intensity distribution. Applying FFT on this matrix to reconstruct the interferogram’s phase distribution is one of the most well-known image processing techniques. A suitable mask can be applied to select a certain peak of the FFT spectrum. This peak represents the power of the number of repetation (i.e. frequency) of a certain behavior of fringes’ distribution. Afterwards, applying the inverse fast Fourier transformation (IFFT) according to the procedure described elsewhere^37,47,48^ to obtain the 2D phase map can be implemented.

In this study, the analyses of the obtained interference patterns and reconstruction of their maps of phase distribution are performed using an algorithm prepared by the aid of “MATLAB” environment. Every phase map is averaged to obtain its unwrapped 1D phase spectrum which is used to calculate the 1D phase difference spectrum between each two successive interferograms. The phase difference (= *2πΔm*) is directly transformed into an optical pathlength difference (i.e. the right hand side of Eq. (3)). Accordingly, the difference in the refractive index can be calculated. The phase shift for any point in each two successive analyzed interferograms must be less than 2π for the image acquisition^54^. Therefore, the recorded frames must be captured with a rate making the phase difference of each point in any two successive frames less than 2π. In this study, adjusting the frames rate as 5 frames/s is found sufficient to detect a phase change between 0 and 2π for any point in the two successive images.

## Results and discussion

**Figure 2:**
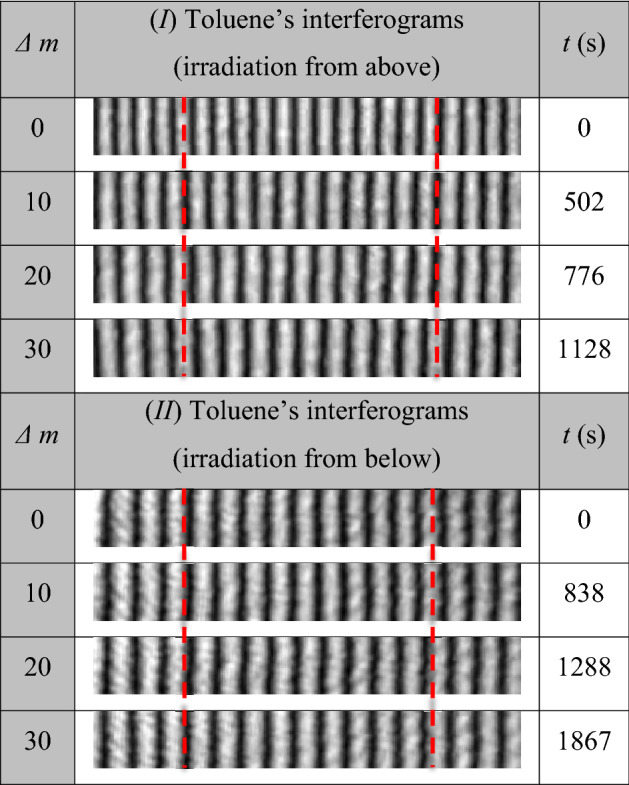
Some interference patterns for toluene at different times of the UV_254_ exposure in case of (I) first and (II) second experimental procedures. The shown *t*-values are the instants when the corresponding *Δm* values are counted.

**Figure 3:**
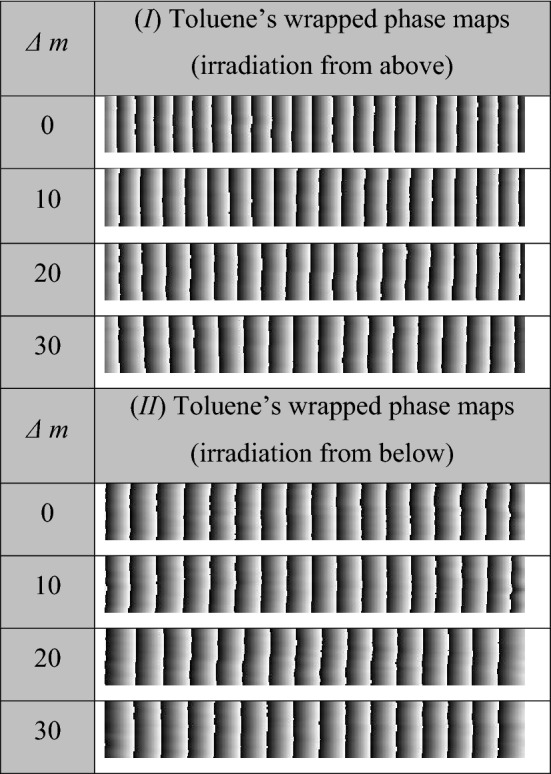
The wrapped phase maps of the interferograms shown in Fig. 2.

**Figure 4:**
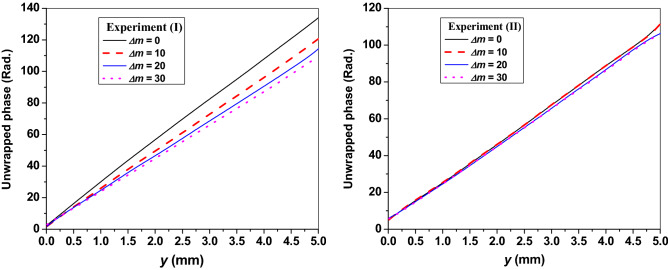
The averaged 1D unwrapped phase profiles of the interferograms shown in Fig. 2.

For the two experimental procedures described in “Methods”, the recorded interferograms where there was no change in the refractive index (i.e., at time of UV_254_ irradiation *t* = 0 s) are illustrated in the first rows in Fig. 2-I, -II). Each interferogram represents a 5 mm distance of equidistant interference fringes. The left side of each interferogram is corresponding to the surface of the sample and is taken as a reference point to monitor the forthcoming changes of refractive index (i.e. movement of interference fringes) upon the UV_254_ irradiation. Some representative consequent interferograms with their corresponding *t* values are illustrated in Fig. 2 for the both cases of UV_254_ irradiation. The shown *t*-values are corresponding to certain values of *Δm*. There is a “written” time value when a number *Δm* = 10 is counted in both experiments*.* The difference in time of two cases is due to the rate of the reaction where the reaction is faster when the sample is UV_254_ irradiated from above. Accordingly, if a number *10 Δm* is counted from the beginning of both experiments, it is found that this number takes place in a shorter time (*t* = 502 s) when the irradiation is from above compared with the longer time (*t* = 838 s) in the other case of irradiation. Based on the FFT analyses of these interferograms^37,47,48^, the reconstructed wrapped phase maps are shown in Fig. 3 while Fig. 4 shows the corresponding averaged 1D unwrapped phase profiles.

From these figures, the fringes’ movement expresses a decrease of refractive index with increasing *t*. In addition to this movement of fringes, it can be noticed that the change in the interfringe spacing for interferograms of case (*I*) represents a noticeable spatial variation in the refractive index through the distance 5 mm at a certain time. Moreover, the rate of interfringe spacing change is faster near the sample’s surface while this rate of change decreases gradually by going inside the sample’s interior. This can be noticed from tracing a certain section in each interferogram and comparing the number of fringes per unit length and their distributions with the time as shown in Fig. 2-I. However, the interfringe spacing of similar monitored distance, in the case of experiment (II), doesn’t have such big difference with increasing *t*. The difference in refractive index from a point to another besides the variation of the same point’s refractive index with time means that the VOC under study exhibits spatio-temporal refractive index variations with the UV_254_ irradiation. Mainly, the temporal variations in the refractive index can be attributed to the photolysis of toluene where the energy of the used UV source is capable of breaking down the C-H bond and the formation of the benzyl radicals^49^. The required energy to break down this bond is 3.89 eV which is easily verified using the UV_254_ source having the energy 4.88 eV. On the other hand, the significant spatial variation, noticed in case (*I*), is due to the oxidation of the benzyl radicals when react with the oxygen molecules (O_2_) exist above the VOC’s surface^50–52^. This amount of O_2_ is more abundant than the amount of soluble O_2_ (in toluene) in case (*II*) and that is why the rate of refractive index variation is slower in case (*II*). Additional information can be found in Appendix 2 where Fourier-transform infrared spectroscopy (FTIR) and UV/VIS absorbance spectra of the studied samples are presented.

**Figure 5:**
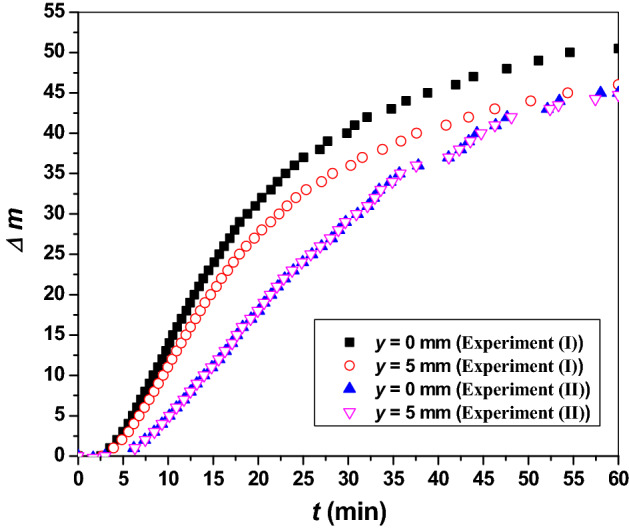
*Δm* as a function of *t* for toluene under the two conditions of the UV_254_ exposure for the points *y* = 0 mm and *y* = 5 mm.

To estimate the spatio-temporal variations of toluene’s refractive index during the whole 60 min of experiment, the relations of *Δm* as a function of *t* in the two procedures of UV_254_ irradiation are obtained. Figure 5 show these relations for the points *y* = 0 mm (i.e., at the surface) and *y* = 5 mm (i.e., the farthest monitored point). This figure reveals that the spatial variation in case (*II*) is significantly low compared with case (*I*).

**Figure 6:**
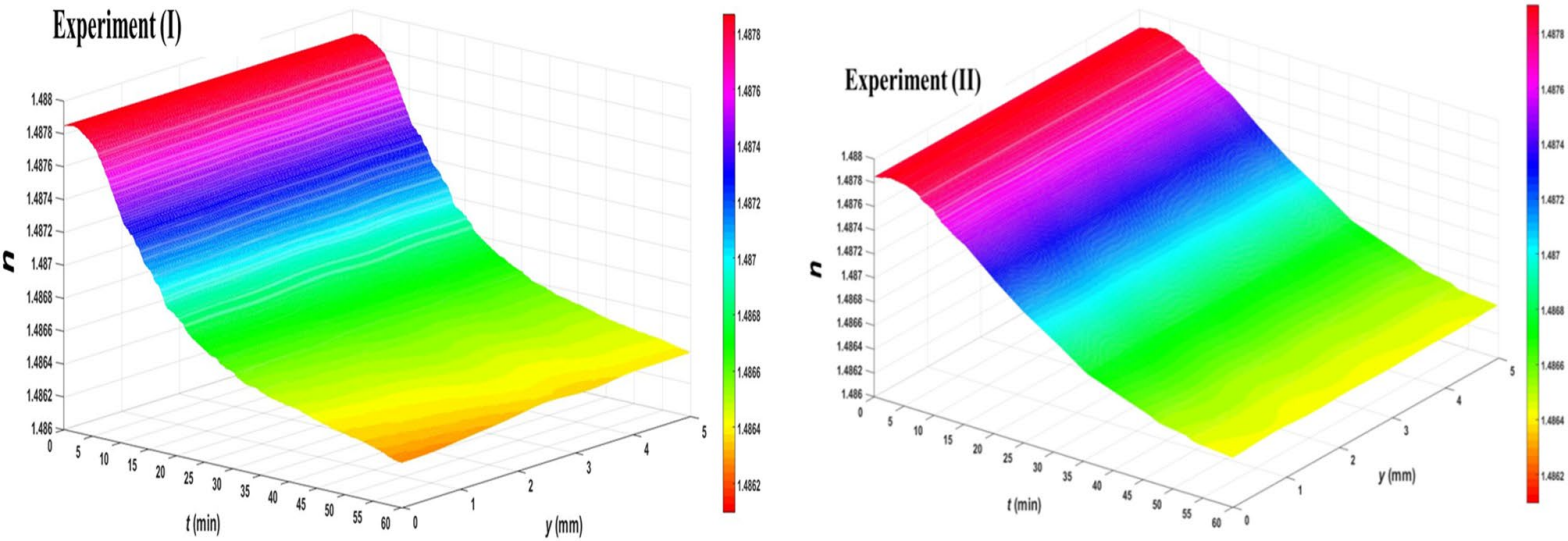
Persistent spatio-temporal refractive index profiles of UV_254_ irradiated toluene for both experiments.

Moreover, Fig. 6 show the toluene’s persistent spatio-temporal refractive index profiles for cases (*I*) and (*II*), respectively. The whole profiles have a decreasing feature with increasing *t*. Furthermore, the refractive index at each *t* value changes with the depth *y* in case (*I*) while its value, barely, fluctuates around an almost constant value for each *t* value in case (*II*). Additionally, with increasing *t*, the spatial profiles have different behaviors. At the beginning of irradiation, the whole variation and the refractive index variation from a point to another are too low. The rate of variation increases at intermediate *t* values while it starts to decrease afterwards. The use of Michelson interferometer which permits passing of the object beam twice through the sample, which is also having a long path (i.e., width of the cuvette), as well as the digital processing of the interferograms provides an accuracy better than 1 × 10^–5^ in the refractive index measurement.

**Figure 7:**
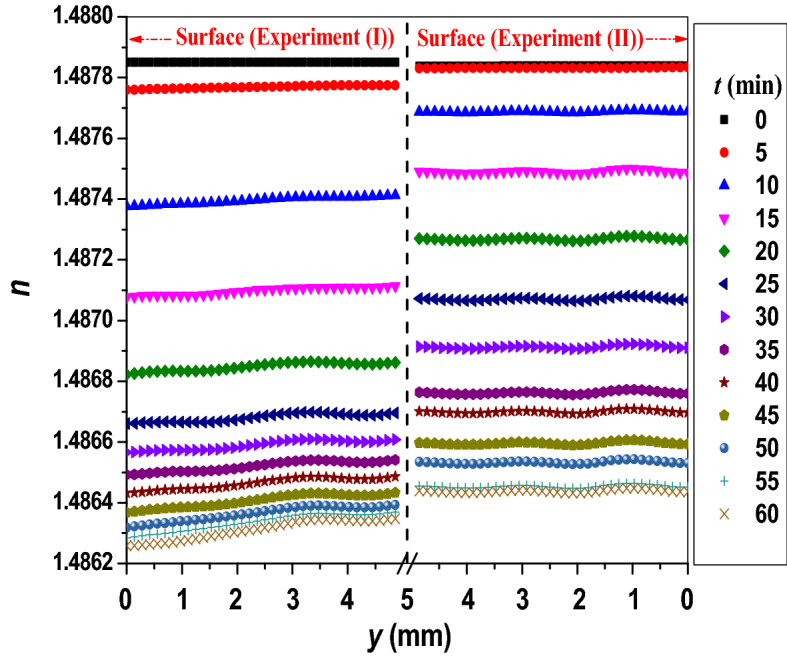
Spatial refractive index profiles of UV_254_ irradiated toluene at discrete times of irradiation in the two experiments.

In order to gain a deeper insight into how the variations are significantly different for the UV_254_ irradiated toluene, discrete refractive index profiles *n*(*y*) (separated by 5 min) are extracted from Fig. 6-I and -II and are plotted in Fig. 7 for comparison.

**Figure 8:**
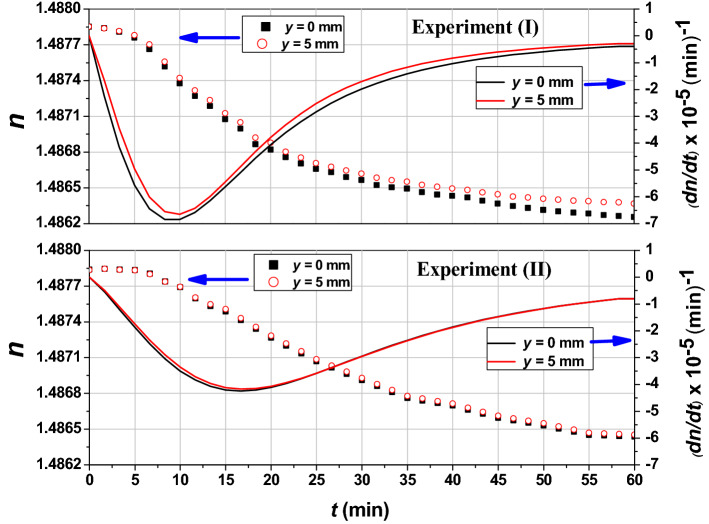
Temporal refractive index profiles of toluene for the points *y* = 0 mm and *y* = 5 mm for the two cases of UV_254_ irradiation.

Actually, a more comprehensive quantification can be deduced from the above figures. Therefore, the curves of *n*(*t*) for, only, the points *y* = 0 mm and *y* = 5 mm in the two cases of UV_254_ irradiation are plotted in Fig. 8. From Fig. 8-I, the difference between each two refractive index values at the same instant increases with increasing *t* from zero to about 1.13 × 10^–4^ at the end of irradiation while it is about an order of magnitude lower in case (*II*). Also, *n*(*t*) in case (*II*) takes longer time to reach its half value (*t*_*0.5*_) due to the less amount of soluble O_2_ in toluene compared with the amount of O_2_ in case (*I*) which, of course, slows down the rate of the chemical reaction.

As a mathematical description, it is found that the behavior of *n* as a function of *t* obeys a logistic decaying behavior which can be expressed by Eq. (4) ^53^.

4 $$n\left(t\right)=\frac{{n}_{initial}-{n}_{final}}{1+{\left(\frac{t}{{t}_{0.5}}\right)}^{p}}+{n}_{final},$$

where, *n*_*initial*_ is the initial value of refractive index (at *t* = 0 min), *n*_*final*_ is the final value of refractive index (at *t* = 60 min) and ${t}_{0.5}$ is the time when *n*(*t*) reaches its half value while *p* is the decaying factor. These values for the four *n*(*t*) curves of toluene, shown in Fig. 8, are illustrated in Table 1. The *R*^2^ values of all fittings are higher than 0.9960 as shown in the table. Interestingly, there are some recent works reported on the degradation of different materials which can be modeled as a logistic decay. For instance, the electric degradation of platinum group metal-free catalysts^54^ and the thermal degradation of polymers^55^ and fibrils of protein^56^.

**Table 1:** Values of the toluene logistic fittings’ constants.

Experiment	*y* (mm)	*n*_*initial*_	*n*_*final*_	${t}_{0.5}$ (min)	*p*	*R*^2^	*t*_*max*_ (min)
I	0	1.48788	1.48616	16.13	1.98	0.9985	10.00
	5	1.48786	1.48633	15.41	2.14	0.9968	10.00
II	0	1.48786	1.48619	26.52	2.15	0.9994	16.67
	5	1.48786	1.48621	26.82	2.18	0.9995	16.67

Additionally, by differentiating the obtained logistic relations with respect to time, the rate of refractive index decrease with increasing *t* (i.e., *dn*(*t*)**/***dt*), which expresses the rate of photolysis, can be obtained. The curves describing this behavior are plotted (as solid curves) on the *n*(*t*) graphs shown in Fig. 8. It is clear that the rate of photolysis is different in both experiments. Moreover, the rate of photolysis, significantly, differs from the surface to the VOC’s interior in case of experiment (*I*). In both experiments, the rate of reaction increases until reaching a certain point and, then, it gets decreased. These turning points represent the maximum variations of *n*(*t*). The values of time corresponding to maximum variations (*t*_*max*_) are determined to be compared with *t*_0.5_ values, see Table 1. The photolysis rate of toluene was recently reported as 2.5** × **10^–5^ s^−1^ (or 1.5** × **10^–3^ min^−1^) and 4.5** × **10^–4^ s^−1^ (or 2.7** × **10^–2^ min^−1^) when it is dissolved in water and is existed at an icy surface, respectively^29,35^. It was found that the rate of photolysis is enhanced due to a red shift of the toluene’s absorbance spectrum at the ice surface. However, these rates (of toluene and other VOCs^29,35,36,57^) were considered constants.

Where they have been calculated as the slopes of “fitted” linear relations of the fluorescence intensity versus time of irradiation graphs. In contrast, the presented study enables a continuously and instantaneously monitoring of the rate of photolysis via monitoring the refractive index dynamic variations and by the aid of an interferometric tool.

At the end, the spatio-temporal variations of refractive index foretells that the UV_254_ irradiated toluene, particularly that is irradiated from above, can be operated as a graded index fluidic waveguide^58–60^ and this will be considered in a future work. Moreover, different characteristics of VOCs have to be explored under different experimental conditions since the experiment is strongly sensitive to the ambient conditions as well as the way of the UV irradiation.

## Conclusions

The refractive index variations of the UV_254_ irradiated toluene are studied under two cases of irradiation; when the surface is, directly, exposed to air and when the surface is not exposed to the air. Michelson interferometer is used to record the interferograms of the studied samples under the effect of the UV_254_ irradiation. The interferograms are analyzed in order to reconstruct their corresponding phase maps which are utilized to calculate the spatio-temporal refractive index profiles of toluene. In the case of absence of air at the surface of toluene, the refractive index in a 5 mm depth decreases temporally with the UV_254_ irradiation. On the other hand, when the surface is exposed to an amount of stagnant air, the refractive index exhibits a more significant spatial variation (i.e. with depth) in addition to the temporal variation. The temporal variation is attributed to a photolysis effect results from breaking of the toluene’s C–H bonds due to the sufficient energy of the used irradiation source, 4.88 eV, and the formation of the benzyl radicals. The spatial variation of refractive index is attributed to the oxidation of benzyl radicals when interacts with the oxygen molecules which are abundant above the toluene’s surface compared with their lower dissolved amount in toluene. The decrease of refractive index with the time of irradiation is modeled as a logistic function. From the logistic fittings, different parameters are obtained; e.g. the time when the refractive index reaches its half value as well as the time at which the photolysis rate reaches its maximum value. This besides the continuous monitoring of the spontaneously rate of toluene’s photolysis; where a photolysis rate in the order of 10^–5^ min^−1^ is detected.

## Supplementary Information

Supplementary Information.

## Data Availability

All data generated or analysed during this study are included in this article.
