# Supplementary material for: Spatio-temporal photolysis rate profiles of UV254 irradiated toluene
Source: Sci Rep. 2022 Jul 26;12:12744. doi: 10.1038/s41598-022-16941-6 (PMC9325976; doi:10.1038/s41598-022-16941-6)
Supplement: Supplementary file 1 — Supplementary Information. [file 41598_2022_16941_MOESM1_ESM.docx]

**Appendix (1)**

To make sure that the variation of refractive index in the presented study is not due to a thermal effect of irradiation, a simple measurement is performed. The temperature of the sample (under both cases of irradiation) is measured with the whole time of irradiation. After one hour of irradiation, the maximum increase in temperature is found to be ca. 0.6 ^o^C. This increase in temperature might be a result of the irradiation where an amount of energy is given to the sample by the UV irradiation.

Since there are new productions of degradation as can be seen from the IR spectra in appendix (2), these productions of degradation can’t be obtained with this trivial amount of thermal energy ^1–4^, i.e., the presented case is not a thermal decomposition.

Additionally, referring to the toluene’s thermo-optic coefficient (*dn*/*dθ*) which is (– 5.55 × 10^-4^ (^o^C^-1^)) at *λ*=632.8 nm and 25 ^o^C ^5,6^, the obtained 0.6 ^o^C increase in temperature would be responsible on a decrease in refractive index 3.3 × 10^-4^ which is about an order of magnitude lower than the already obtained decrease in refractive index due the UV_254_ irradiation as shown in figure (7) in the manuscript.

**Appendix (2)**

Fourier-transform infrared spectroscopy (FTIR) transmittance spectra of the un-irradiated and the UV_254_ irradiated (for 1 hour) toluene are presented for comparison. A Jasco-spectrometer (FT/IR-4100)) is used. The sample which is irradiated from above (i.e., in the presence of oxygen at the surface) shows two peaks at wavenumbers (1280 and 1740) cm^-1^ meaning the formation of C-O and C=O, respectively ^7–9^. On the other hand, the sample which is irradiated from the bottom has, almost, the same spectrum of the un-irradiated one except a very weak peak at the wavenumber 1280 cm^-1^ corresponding a barely formation of C-O bond, see the following figure.

Moreover, the UV/VIS spectra of the same studied samples are performed using a Jasco-spectrophotometer (V-630). The UV/VIS spectrum of the sample which is irradiated from above suffers a broadening as can be seen in the following figure. This, also, can explain the appearance of the two peaks in the FTIR spectrum of this sample. On the other hand, the sample which is irradiated from the bottom has, almost, the same spectrum of the un-irradiated one but with higher amplitude. It has been reported that the absorbance of degraded toluene increases under the effect of UV irradiation ^2,3^.

**References**

1. Hermans, I., Peeters, J., Vereecken, L. & Jacobs, P. A. Mechanism of Thermal Toluene Autoxidation. *ChemPhysChem* **8**, 2678–2688 (2007).

2. Oehlschlaeger, M. A., Davidson, D. F. & Hanson, R. K. Thermal decomposition of toluene: Overall rate and branching ratio. *Proceedings of the Combustion Institute* **31**, 211–219 (2007).

3. Astholz, D. C., Durant, J. & Troe, J. Thermal decomposition of toluene and of benzyl radicals in shock waves. *Symposium (International) on Combustion* **18**, 885–892 (1981).

4. Brouwer, L. D., Mueller-Markgraf, W. & Troe, J. Thermal decomposition of toluene: a comparison of thermal and laser-photochemical activation experiments. *J. Phys. Chem.* **92**, 4905–4914 (1988).

5. Hauf, W. & Grigull, U. Optical Methods in Heat Transfer. in *Advances in Heat Transfer* vol. 6 133–366 (Elsevier, 1970).

6. Weber, M. J. *Handbook of optical materials*. (CRC Press, 2003).

7. Alpert, N. L., Keiser, W. E. & Szymanski, H. A. *Ir Theory and Practice of Infrared Spectroscopy.* (Springer, 1995).

8. Larkin, P. *Infrared and raman spectroscopy: principles and spectral interpretation*. (Elsevier, 2011).

9. https://www.sigmaaldrich.com/EG/en/technical-documents/technical-article/analytical-chemistry/photometry-and-reflectometry/ir-spectrum-table.
